# Editorial: Spectator sport and fan behavior: a sequel

**DOI:** 10.3389/fpsyg.2024.1414407

**Published:** 2024-05-02

**Authors:** Yair Galily, Simon Pack, Ilan Tamir

**Affiliations:** ^1^Ivcher School of Psychology, Reichman University, Herzliya, Israel; ^2^The Lesley H. and William L. Collins College of Professional Studies, St. John's University, Queens, NY, United States; ^3^The Moskowitz School of Communication, Ariel University, Ariel, Israel

**Keywords:** media, AI, television, soccer, e-sport

In the grand amphitheaters of modern society, spectator sports emerge as vibrant tapestries that weave together the passions, emotions, and collective spirit of millions (Galily, 2019; Galily et al., 2023). From the thunderous roars of a stadium to the intimate hum of a neighborhood bar, the allure of spectator sports transcends geographical, cultural, and social boundaries. At the heart of this phenomenon lies the intricate dance between the athletes on the field and the fervent supporters in the stands. The dynamic relationship between spectator sport and fan behavior is a captivating study, evolving around psychology, sociology, and anthropology that shape this unique and powerful connection (Dwyer et al., 2018).

Spectator sports, ranging from football and basketball to cricket and rugby, have become modern-day rituals, fostering a sense of identity and community among diverse groups of people. As fans gather in colossal arenas or huddle around television screens, they embark on a collective journey fueled by a shared love for the game. The spectacle unfolds not only on the playing field but also in the stands, where the energy of the crowd becomes an integral part of the sporting experience.

The behavior of sports fans is a multifaceted phenomenon that defies easy categorization (Giulianotti, 2002). It encompasses a broad spectrum, ranging from the exuberant celebration of triumph to the despondent anguish of defeat. Fans invest emotionally, mentally, and even financially in their chosen teams, transforming a mere pastime into a visceral, sometimes all-consuming, passion. This deep emotional involvement often transcends reason, as the victories and losses of a team mirror the highs and lows of the fans' own lives.

The psychology of fan behavior is a captivating subject that psychologists and sociologists have probed for decades. The concept of sports fandom taps into fundamental human instincts, such as the desire for belonging and the need to identify with a collective. Fans often see their chosen teams as extensions of themselves, forging a powerful connection that goes beyond the boundaries of the playing field. This psychological investment influences how fans interpret events, form allegiances, and even navigate the complexities of their own social identities.

Beyond the emotional investment, spectator sports have also become arenas for social interaction and cultural expression. Stadiums and sports bars transform into communal spaces where diverse individuals unite under the banner of shared allegiance. The ritualistic nature of sports fandom, from pre-game tailgates to post-victory celebrations, creates a sense of camaraderie that transcends societal divisions. These shared experiences become threads in the fabric of cultural identity, contributing to the rich tapestry of human social interaction.

However, the euphoria of fandom is not without its darker shades. The passionate devotion to a team can sometimes manifest in aggressive or unruly behavior. Instances of hooliganism, verbal abuse, and even violence are stains on the otherwise colorful canvas of sports fandom. Understanding the factors that contribute to such extremes is crucial in developing strategies to ensure that the communal joy of sports does not devolve into a chaotic and unsafe environment.

In this exploration of spectator sport and fan behavior, we unraveled the intricate threads that compose this complex tapestry. From the psychological underpinnings of fandom to the societal impact of sports culture, our journey delved into the heart of this phenomenon, examining the highs and lows, the unity and discord, and ultimately, the enduring allure that makes spectator sports an integral part of the human experience.

Eleven papers included in our current Research Topic. Lev et al. study sheds light on the dialogue between older players and the audience. Similarly, Kaden et al. paper focuses on German soccer fans because an increasing number of media reports note problems for the fans in this regard, reporting eroded loyalty, increasing alienation, or turning away. Galily et al. research examined a variety of demographic factors influencing browsing device trends before, during (“second screen”), and after sports games. It does so by utilizing survey data from Israeli viewers of the 2022 world cup using a convenience sample (*n* = 242). Still with the world cup, Deng et al. analysis explored online fan satisfaction with the video assistant referee (VAR) during the FIFA world cup Qatar 2022. A structural equation model comprising perceived value, fan expectation, fan identification, and fan satisfaction. Thereafter, Schleifer and Tamir article focuses on the Esthetics of team uniform Colors and identifies the artistic roles they fill. Shi and Ren study identified the motivations that relate to Chinese e-sports viewers' attitude as well as their watching intention, and underscores the role of satisfaction with past experience, which were understudied. Ryu et al. research aims to explore the determinants of the league of legends champions Korea highlight views and comment counts. The data of 629 game highlight views and comment counts for seven tournaments were collected from YouTube. Next, Levental et al. study presents a thorough investigation of the attitudes and emotions expressed by the spouses and girlfriends of sports fans within the context of their long-term relationships.

Within online communities, Levental conceptual examination used several examples of online sports fan communities in Israel to analyze their core elements. Within this context, the article focuses on the significance of humor, trivial knowledge, and counter-modern concepts as the key elements fostering unity among fans. Then, Glebova et al. tried to answer the question “How (and why) do sports venue digital twin emerging technologies prospectively impact the sports spectators” customer experiences?” Lastly, Hayat enquired about the dynamics between online social connections and creative expressions in the realm of ESports. Therefore, this research paper examined a correlation between people's creativity and their Effective Network Size (non-redundant ties) on Twitter, to see if potentially non-redundant information is related to creativity.

Indeed, further research on spectator sport and fan behavior is imperative to comprehensively understand the intricate dynamics at play. Future key areas requiring investigation include the psychological motivations behind fan behavior, social dynamics within fan communities, the impact of technology on fan engagement, instances of fan violence and aggression, the economic implications of spectator sports, cultural influences on fan behavior, and the efficacy of fan engagement strategies. By delving deeper into these areas, researchers can contribute valuable insights that inform the development of policies and practices aimed at enhancing the spectator experience, fostering fan loyalty, and ensuring the long-term viability of spectator sports. Additionally, integrating AI methodologies into research on spectator sport and fan behavior could provide novel insights by analyzing vast datasets, predicting fan trends, and personalizing fan experiences, thus further advancing our understanding of this complex phenomenon (Galily, 2018).

## Author contributions

YG: Conceptualization, Methodology, Writing—original draft. SP: Conceptualization, Formal analysis, Writing—review & editing. IT: Formal analysis, Writing—original draft.
